# Route of infectious bronchitis virus vaccination determines the type and magnitude of immune responses in table egg laying hens

**DOI:** 10.1186/s13567-021-01008-7

**Published:** 2021-11-12

**Authors:** Mohammed Al-Rasheed, Christopher Ball, Kannan Ganapathy

**Affiliations:** 1 Institute of Infection, Veterinary and Ecology Sciences, University of Liverpool, Cheshire, UK; 2 College of Veterinary Medicine, King Faisal University, Al-Ahsa, Saudi Arabia; 3 Avian Research Center, King Faisal University, Al-Ahsa, Saudi Arabia

**Keywords:** Avian coronavirus, infectious bronchitis virus, vaccination, layer chicken, gene transcription, innate, mucosal, cellular and humoral immunity

## Abstract

Chicken immune responses to infectious bronchitis virus (IBV) vaccination can depend on route of administration, vaccine strain and bird age. Typically for layer chickens, IBV vaccinations are administered by spray in the hatchery at day-old and boosted at intervals with live vaccines via drinking water (DW). Knowledge of live attenuated IBV vaccine virus kinetics and the immune response in egg-laying hens is exceptionally limited. Here, we demonstrated dissemination of vaccine viruses and differences in hen innate, mucosal, cellular and humoral immune responses following vaccination with Massachusetts or 793B strains, administered by DW or oculonasal (ON) routes. Detection of IBV in the Mass-vaccinated groups was greater during early time-points, however, 793B was detected more frequently at later timepoints. Viral RNA loads in the Harderian gland and turbinate tissues were significantly higher for ON-Mass compared to all other vaccinated groups. Lachrymal fluid IgY levels were significantly greater than the control at 14 days post-vaccination (dpv) for both vaccine serotypes, and IgA mRNA levels were significantly greater in ON-vaccinated groups compared to DW-vaccinated groups, demonstrating robust mucosal immune responses. Cell mediated immune gene transcripts (CD8-α and CD8-β) were up-regulated in turbinate and trachea tissues. For both vaccines, dissemination and vaccine virus clearance was slower when given by DW compared to the ON route. For ON administration, both vaccines induced comparable levels of mucosal immunity. The Mass vaccine induced cellular immunity to similar levels regardless of vaccination method. When given either by ON or DW, 793B vaccination induced significantly higher levels of humoral immunity.

## Introduction

Commercial laying hens infected with infectious bronchitis virus (IBV) can exhibit both respiratory [1] and reproductive system [2, 3] complications. Oviduct infection can lead to reduced laying production and lower egg quality [4–7], resulting in economic losses for poultry farmers. In the United Kingdom (UK), infectious bronchitis (IB) affected approximately 22.5 million chickens at an estimated total cost of £12.6 million from egg production losses between 2005 and 2019 [8, 9]. Egg quality problems include reduced shell thickness, a mottled and discoloured shell, and watery albumin [10]. Prevention of infectious bronchitis (IB) in egg-laying chickens is mainly through the use of inactivated and live vaccines during the rearing period, and the vaccine strains administered are dependent on the prevalent endemic strains [11].

Live attenuated IBV vaccines are used to induce local immunity, which is important as a part of an effective host mucosal immune response [12–14]. Live vaccines are administered to day-old chicks and then at intervals, to achieve early and continuous mucosal protection. At 4–6 weeks before the onset of lay, each bird also receives an inactivated vaccine to boost humoral antibody levels [15–17]. The gold standard approach for IBV vaccination is oculonasal (ON) [18, 19], however, this route is not practical for vaccine administration to a large number of birds. For this reason, in the field, live vaccines are typically administered via drinking water [20, 21], coarse spray [22] or aerosols at day-old or within the first week of age [19]. Though publications are available on the assessment of live vaccines in young birds, to date, there has not been any report on the impact of administering monovalent vaccines in older layer hens.

The Massachusetts (Mass) serotype vaccine was the first to be produced, and strains belonging to Mass (such as H120 and Ma5) are used throughout the world for laying hens [23–25]. Another serotype that is widely distributed in Europe, Asia, and other parts of the world is 793B [26]. Strains identified as the 793B serotype, including 793B, CR88 and 1/96, emerged in the 1990s and have been associated with welfare and economic problems in flocks [27, 28].

The innate, mucosal, cellular and immune responses are vital for protection against virulent IBVs. In terms of early immunity following IBV vaccination, previous work has outlined transcriptional expression of toll-like receptors (TLRs), melanoma differentiation-associated protein 5 (MDA5), type I interferons, and pro-inflammatory cytokines [14, 29].

While previous studies have investigated specific-pathogen-free (SPF) or broiler chickens, to date, there have been no publications investigating host immune responses in the respiratory or head-associated lymphoid tissues (HALT) of egg-laying hens following IBV vaccination. We report vaccine virus distribution in a number of tissues, and the host immune responses in 41-week-old egg-laying hens following administration of Mass (Mass) or 793B live vaccines, either by oculonasal or drinking water routes.

## Materials and methods

### Ethical statement

All experimental procedures were performed according to the UK legislation governing experimental animals under the project licence P8E4FC2C9. Experimental procedures were approved by the University of Liverpool’s ethical review process.

### Layer chickens

Seventy-two 41-week-old light brown Lohman layer chickens were obtained from a commercial UK farm and kept up to three weeks under strict biosecurity measures in the University of Liverpool. When these birds were in the rearing farm, the flock received live IBV Massachusetts vaccination at day-old, followed by a live 793B vaccine at day-14. Subsequently, an inactivated IBV M41 +D274 was given at 15 weeks of age. No IB or other vaccines were applied during the laying period. For this study, birds were transported to University of Liverpool experimental house one week prior to vaccination to allow for acclimatisation to conditions. Hens were reared on deep litter with nest boxes, and bird-friendly toys, antibiotic-free water and feed were provided ad libitum.

### Attenuated live IBV vaccines

Two commercial live IBV vaccines, Massachusetts (Mass) serotype (H120, Boehringer Ingelheim Animal Health Limited, UK) and 793B serotype (4/91, MSD Animal Health, UK) were prepared according to manufacturer’s instructions. One vial of each vaccine was thoroughly mixed in 100 mL of chilled distilled water and kept on crushed ice before use. Each hen received 100 μL of the vaccine or sterile distilled water (SDW) via the oculonasal (ON) route. For the drinking water (DW) group, to ensure uptake of full vaccine dosage, the vaccine was administered orally. The dosage was maintained as administered in commercial farms. Vaccine strains were titrated in tracheal organ cultures (TOCs) to determine the mean tissue culture infectious dose (TCID_50_); Mass (10^3.75^ TCID_50_/mL) and 793B (10^4.45^ TCID_50_/mL) [30].

### Experimental design

Oropharyngeal (OP) swabs were collected one week before the hens arrived at the University (40 weeks of age) to confirm an absence of IBV by RT-PCR. Upon arrival, seventy two 41-week-old layer chickens were divided into six groups (*n*  = 12 per group); (A1) DW-unvaccinated control, (A2) ON-unvaccinated control, (B1) DW-Mass, (B2) ON-Mass, (C1) DW-793B and (C2) ON-793B. Prior to vaccination, egg production and clinical signs were monitored for one week. At 42-weeks-old, OP swabs were collected from 10 birds in each group to confirm absence of IBV, avian metapneumovirus (aMPV), *Mycoplasma gallisepticum* and *Mycoplasma synoviae* by PCR. Groups A1 and A2 were sham-inoculated with 0.1 mL of vaccine-free SDW. Groups B1, B2, C1 and C2 were vaccinated with 0.1 mL of live Mass (10^3.75^ TCID_50_/mL) or 793B (10^4.45^ TCID_50_/mL). Following vaccination, OP and cloacal (CL) swabs were collected from five chickens at 1, 3, 5, 7, and 14 dpv for virus detection and quantification by quantitative real-time RT-PCR (qRT-PCR). At 7 and 14 dpi, lachrymal fluid and blood were collected from five birds in each group to assay for anti-IBV antibodies by indirect ELISA. Three birds from each group were humanely euthanized at 1, 3, 5, and 14 dpv. The Harderian gland, turbinate, choanal cleft, trachea, caecal tonsil and kidneys were collected and stored at −20 °C in RNALater™ (Qiagen, Crawley, UK) for quantification of viral load and/or host gene expression analysis by qRT-PCR.

### Humoral immune responses by indirect ELISA

Sera were analysed using a commercial IBV ELISA kit (IDEXX, Westbrook, Maine, USA) to determine anti-IBV antibodies according to the manufacturer’s guidelines. Antibody titres were determined by converting the sample/positive ratio according to a formula provided by the manufacturer, with a positive ELISA titre cut-off determined as 396.

### Mucosal immune responses by indirect ELISA

Lachrymal fluid was assayed for IBV-specific IgA and IgY using an indirect monoclonal ELISA [31–33]. Each well of a flat bottom 96-well microplate (STARLAB^®^, UK) was coated with 100 µL of purified 2.5 µg/mL IBV M41 antigen in 50 mM sodium carbonate/bicarbonate buffer (pH 9.6). Plates were incubated for 1 h at 37 °C, and then overnight at 4 °C. Wells were blocked with 200 µL phosphate buffer saline (PBS) containing 3% non-fat skimmed milk powder. Lachrymal fluid samples were tested in triplicate at a single dilution of 1:10 in PBS containing 0.05% tween-20 (PBST) (Sigma Aldrich^®^, Dorset, UK). Mouse monoclonal antibodies against either chicken IgA or IgY (BIO-RAD^®^, Hertfordshire, UK) were added at a dilution of 1:1000 (50 µL) as the secondary antibody, and incubated for an hour at 37 °C. This was followed by goat anti-mouse IgG horse-radish peroxidase-conjugate (BIO-RAD^®^) at a dilution of 1:10 000 (50 µL), and 1 hour incubation at 37 °C. Tetramethylbenzidine (TMB) (Sigma Aldrich^®^) substrate was added to each well (50 µL) and incubated in the dark for 15 min to allow for colour development. The reaction was stopped by adding 50 µL of sodium hydrochloric acid (0.5 M HCL), and plates were analysed at 450 nm. Corrected optical density (COD) values were calculated by deducting the OD values of non-antigen coated (blank) wells for each sample [31, 34].

### Quantification of viral load from swabs and tissues

Viral RNA was extracted from the swab and tissue samples, using the QIAamp viral RNA mini kit and the RNeasy Mini kit (Qiagen, UK) respectively, according to manufacturer’s instructions. Quantification of viral RNA was carried out by qRT-PCR, using an IBV 3’ untranslated region (UTR) gene-specific primer and probe as previously described [35]. Obtained Ct values were converted to log relative equivalent units (REU) of viral RNA by a standard curve generated from using five ten-fold dilutions of RNA extracted from M41 virus-positive allantoic fluid [36, 37].

### Measurement of host gene transcription

Extracted RNA was tested by qRT-PCR for expression of pro-inflammatory cytokine IL-6, innate immune pattern recognition receptors (TLR3 and MDA5), interferon beta (IFN-β) [36–38], mucosal immune responses (IgA and IgY) and cellular immune responses (CD8-α and CD8-β) [14, 39, 40]. Each cDNA sample was tested in triplicate using LightCycler 480 SYBR Green I Master mix and gene specific primers (Table 1). For IL-6, TLR3, MDA5 and IFN-β, data was normalized using a relative standard curve method to 18S ribosomal RNA expression [41] and data presented as the log_2_ fold difference in gene expression of vaccinated against control samples. For IgA, IgY, CD8-α and CD8-β, data was normalized against 18S, and the fold change was calculated using the double delta Ct (∆∆Ct) method. Significant up-regulation or down-regulation was reported when compared with the control group, unless otherwise stated.

**Table 1:** **Details of primers used in the qRT-PCR analysis of gene signatures, interferons and cytokines**

Gene group	Gene target	Primer sequences: forward (F) and reverse (R)	Reference
Reference gene	18S ribosomal RNA	(F) TGTGCCGCTAGAGGTGAAATT	[14]; [41]
		(R) TGGCAAATGCTTTCGCTTT	
Cellular immune response	CD8-α	(F) TTG GAC GGG ACC TTA CAG AC	[14]
		(R) TGA AGG GAG CAA AGG AGA AA	
	CD8-β	(F) CTGCATGGCTCCGACAATGG	[40]
		(R) ATCGACCACGTCAAGCTGGG	
Mucosal immune response	ChIgA (chicken immunoglobulin A)	(F) TGCAGGGCAATGAGTTCGTCTGTA	[42]
		(R) AGGAGGTCACTTTGGAGGTGAAT	
	ChIgY (chicken immunoglobulin G)	(F) GACGAAGCTT TTCCTCTTCT	[43]
		(R) CCCGATTGTA CCCTCTATCG	
Viral recognition	TLR3 (toll-like receptor 3)	(F) GCAATTTCTCCTTCACCTTTTCA	[44]
		(R) CCTTTATGTTTGCTATGTTGTTATTGCT	
	MDA5 (melanoma differentiation-associated protein 5)	(F) AGGAGGACGACCACGATCTCT	[44]
		(R) CCACCTGTCTGGTCTGCATGT	
Interferon	IFN-β [interferon beta (type I)]	(F) TCCAACACCTCTTCAACATGCT	[44]
		(R) TGGCGTGTGCGGTCAAT	
Inflammation	IL-6 (interleukin 6)	(F) CACGATCCGGCAGATGGT	[44]
		(R) TGGGCGGCCGAGTCT	

### Statistical analysis

Data were confirmed to be normally distributed and analysed using GraphPad™ Prism version 6.00. Significant differences between groups were analysed using univariate ANOVA, along with the homogeneity of variance test, to confirm statistical differences within the data set, followed by post hoc Tukey’s testing to compare between each group. When groups had *p*  < 0.05 for the homogeneity of variance test, Tamhane’s T2 was applied post hoc instead of Tukey’s. For IgA, IgY, CD8-α and CD8-β gene transcript fold change comparisons, vaccinated groups were compared to the control group using the *t* test. Differences between groups were considered significant at *p*  < 0.05 unless otherwise stated.

## Results

### Humoral immunity: IBV-specific ELISA

**Figure 1:**
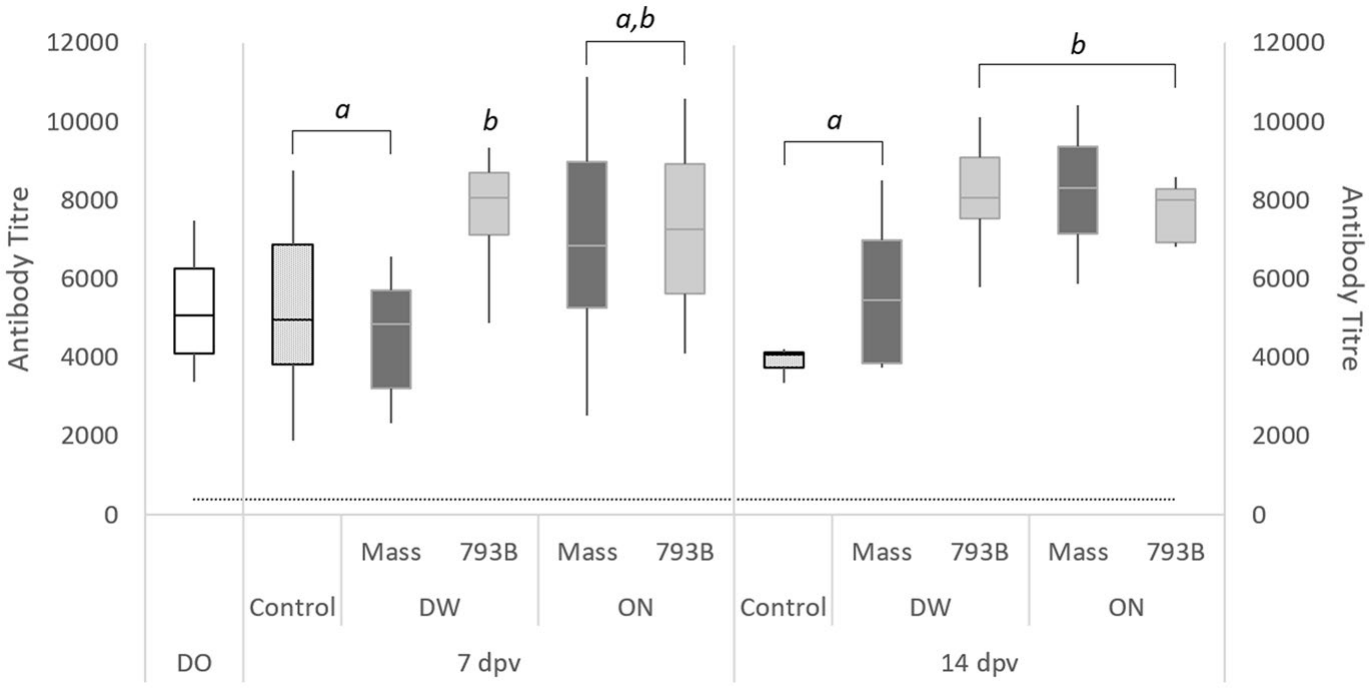
**Mean anti-IBV ELISA antibody titres of the control (*n*   = 8) and vaccinated groups (** ***n***  **= 5) at 0, 7 and 14 dpv**. Data are expressed as mean values  ±  SEM. The positive cut-off titre of 396 is indicated with a dotted line. Different letters indicate significant differences (*p*  < 0.05) within time points, determined using one-way ANOVA. D0:   Day 0; DW:  Drinking water; ON:  Oculonasal.

At 7 dpv, a significantly higher titre was observed in the DW-793B group (8065 ± 792) in comparison with the control (4970 ± 759) and DW-Mass groups (4970 ± 759) (Figure 1). For DW vaccinated birds at 14 dpv, there was a significantly higher antibody titre in the 793B group (8076 ± 556) compared with the Mass-vaccinated (5465 ± 762) and control (4062 ± 218) groups. Significantly higher antibody titres were observed at 14 dpv in ON vaccinated hens [Mass (8306 ± 636) or 793B (7999 ± 461)] compared to the DW-Mass (5465 ± 762) and control (4062 ± 218) groups (Figure 1). No significant differences were noted with the same groups between both sampling days.

### Mucosal immunity: IBV-specific lachrymal IgA and IgY titres

**Figure 2:**
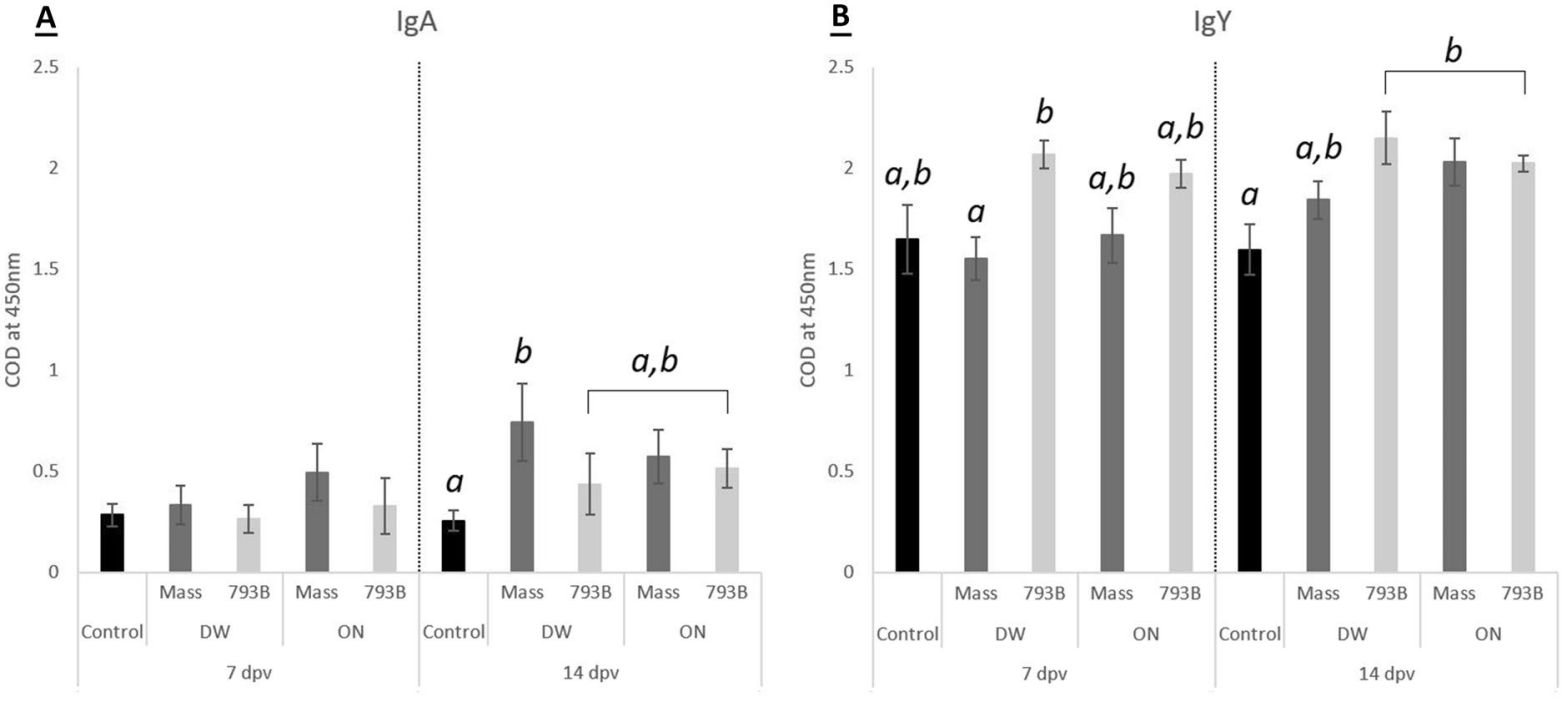
**Measurement of IBV-specific A IgA and B IgY titres using indirect monoclonal ELISA from lachrymal fluid**. Data are expressed as mean of corrected optical density (COD)  ±  SEM. Different letters indicate significant differences (*p * < 0.05) within time points, determined using one-way ANOVA.

Significantly higher IBV-specific IgA levels compared to the control (0.256 ± 0.05) were only observed in the DW-Mass group (0.744 ± 0.19) at 14 dpv (Figure 2A), or when compared to the DW-Mass group at 7 dpv (0.334 ± 0.09). In 793B-vaccinated hens (DW and ON) and ON-Mass vaccinated birds, IgY titres were significantly higher at 14 dpv compared to the control group. For DW-vaccinated groups, the average IgY titre in the 793B group (2.07 ± 0.07) was significantly higher in comparison to the Mass group (1.55 ± 0.1) at 7 dpv (Figure 2B).

### Mucosal immunity: total mRNA expression of IgA in the turbinate and trachea

#### Turbinate

**Figure 3:**
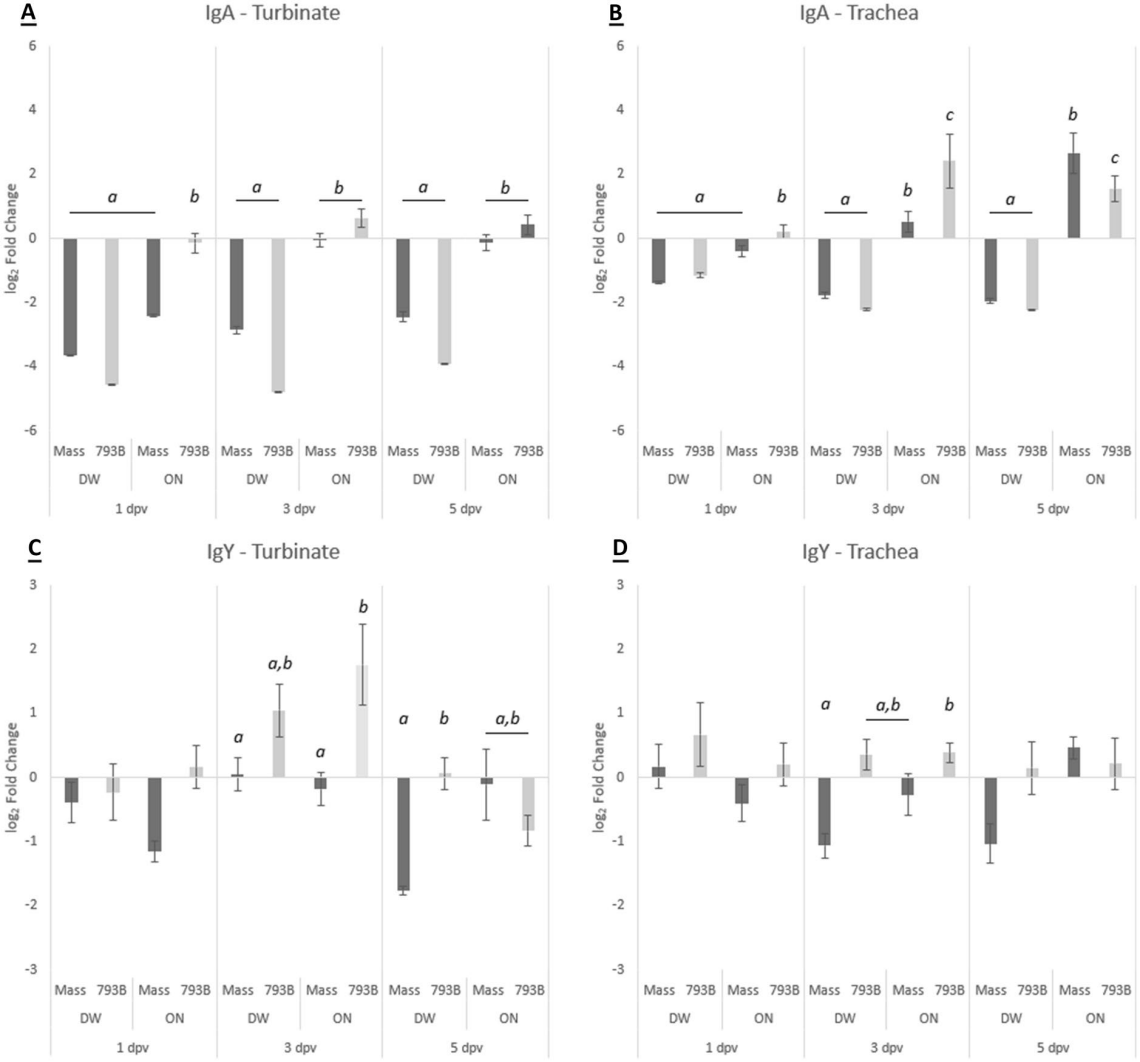
**Relative mRNA expression of IgA in A turbinate and B tracheal tissues, and IgY in C turbinate and D trachea tissues at 1, 3, and 5 dpv**. Data are expressed as log_2_ mean fold change compared to the control group  ±  SEM. Expression is calculated based on double delta Ct (ΔΔCt) values. Different letters indicate significant differences (*p*  <  0.05) within time points, determined using one-way ANOVA.

In both ON-vaccinated groups, there was significant up-regulation in the mRNA expression of IgA at all-time points compared with DW-vaccinated chickens, with the exception of DW-Mass at 1 dpv. In the ON-793B group, there was significant up-regulation of IgA mRNA expression at 1 dpv compared to the ON-Mass vaccinated group (Figure 3A). All DW-vaccinated groups were significantly down-regulated compared with the control, whereas only the ON-Mass group was down-regulated at 1 dpv, with no other differences noted.

#### Trachea

There was significant up-regulation of IgA mRNA expression in the trachea of both ON-vaccinated groups at 3 and 5 dpv, and only at 1 dpv in the ON-793B vaccinated chickens, compared to DW-vaccinated hens. In the ON-793B group, there was significant up-regulation of IgA mRNA expression at 1 and 3 dpv compared to ON-Mass vaccinated group. At 5 dpv, expression of IgA mRNA was significantly higher in the ON-Mass group compared to the ON-793B vaccinated birds (Figure 3B). All DW groups were significantly down-regulated compared to the control, whereas the ON-793B group was significantly up-regulated at 3 and 5 dpv.

### Mucosal immunity: total mRNA expression of IgY in the turbinate and trachea

#### Turbinate

IgY mRNA expression at 3 dpv was significantly higher in the ON-793B-vaccinated group in comparison to both Mass-vaccinated groups. Moreover, expression of IgY mRNA was significantly greater at 5 dpv in 793B-DW birds compared with the Mass-DW group (Figure 3C). At 1 and 5 dpv, the ON-Mass and DW-Mass groups were significantly down-regulated compared with the control respectively, whereas both 793B groups were significantly up-regulated at 3 dpv.

#### Trachea

At 3 dpv, IgY transcripts in the DW-Mass group were significantly lower than the ON-793B group. No other statistical differences were noted (Figure 3D). At 3 and 5 dpv, the DW-Mass group was significantly down-regulated compared to the control.

### Viral RNA quantification: swabs and tissues

#### Individual OP swabs

**Figure 4:**
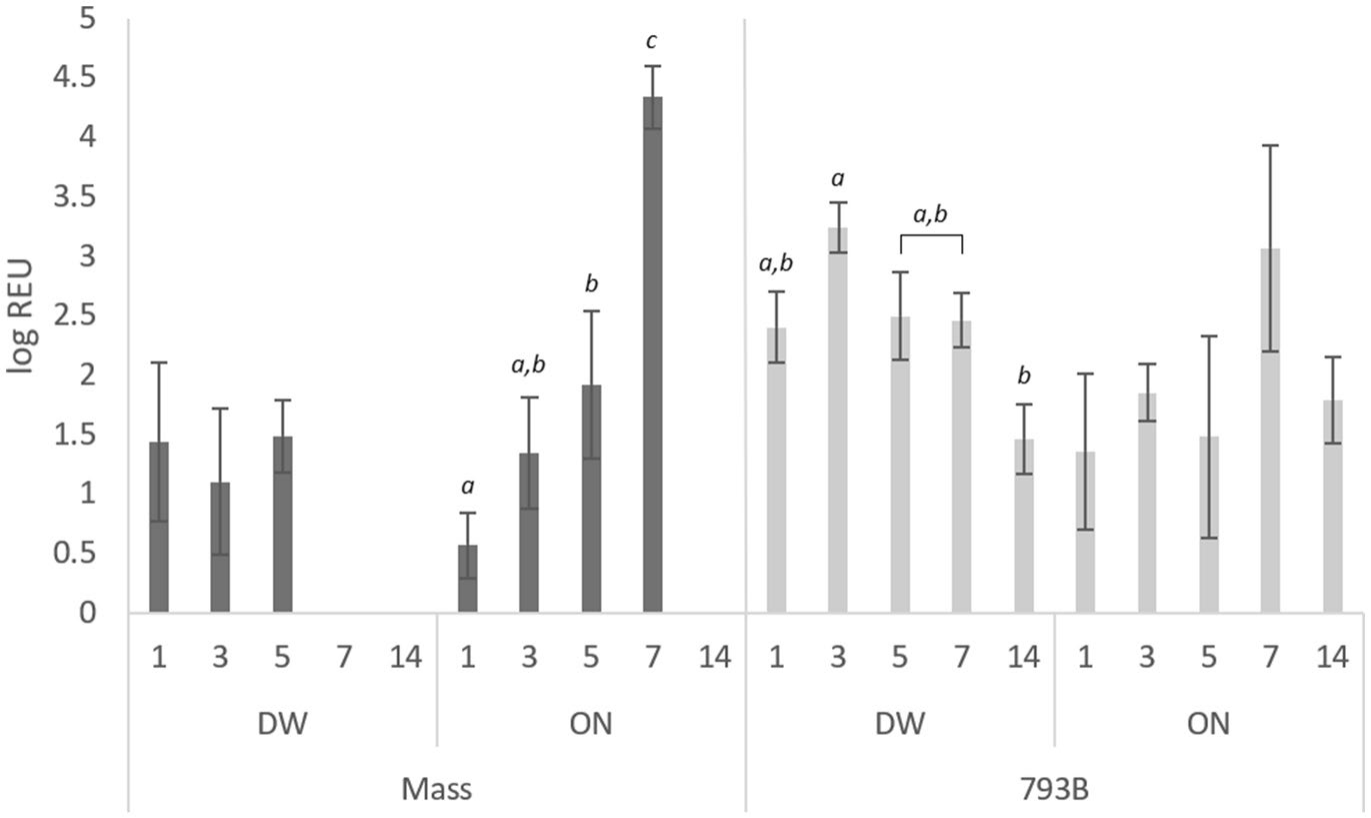
**Quantification of viral RNA from OP swabs, expressed as log-relative equivalent units (REUs) of RNA.** Samples were taken at 1, 3, 5, 7, and 14 dpv. Data presented as the mean  ±  SEM. Different letters indicate significant (*p*  < 0.05) differences within groups.

Viral RNA loads in OP swabs in the control group were below the detection limit. For vaccinated groups, RNA load peaked at 3 dpv for DW-793B birds (3.25 log REU), while the viral RNA load in both ON vaccinated groups peaked at 7 dpv (Mass  = 4.34 log REU; 793B  = 3.07 log REU) (Figure 4). A significant increase was identified from 5 to 7 dpv in the ON-Mass group, with a significant decrease seen in the DW-793B group from 3 to 14dpv.

#### Individual CL swabs

**Figure 5:**
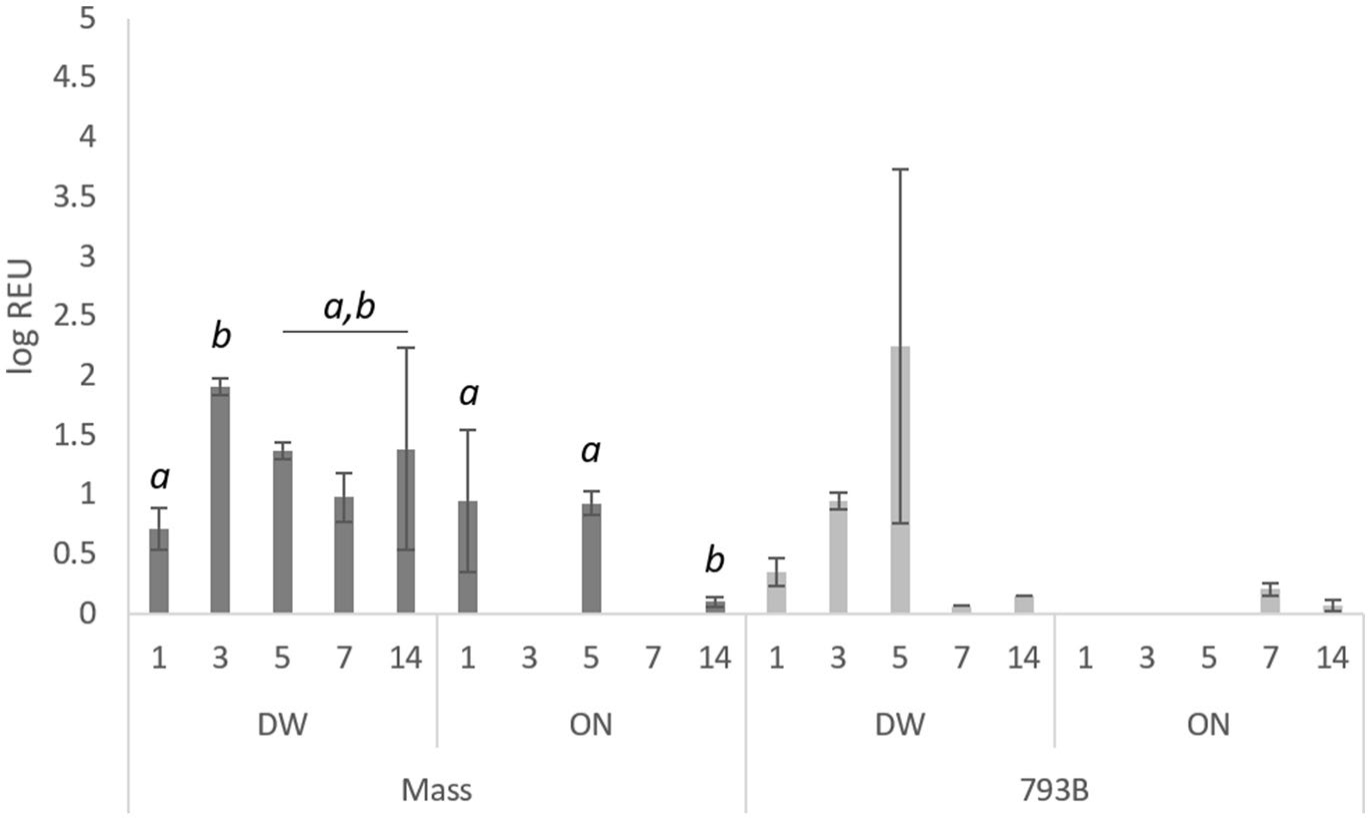
**Quantification of viral RNA from CL swabs, expressed as log-relative equivalent units (REUs) of RNA.** Samples were taken at 1, 3, 5, 7, and 14 dpv. Data presented as the mean  ±  SEM. Different letters indicate significant (*p*  < 0.05) differences within groups.

Viral RNA loads in all CL swabs were below the detection limits in the control group. RNA levels peaked at 3 dpv in the Mass-DW group (1.911 log REU) and 5 dpv in the 793B-DW group (2.249 log REU). There was a significant increase in viral load from 1 to 3 dpv in the Mass-DW group, and a significant reduction from 5 to 14 dpv in the Mass-ON group (Figure 5).

#### Harderian gland (HG)

**Figure 6:**
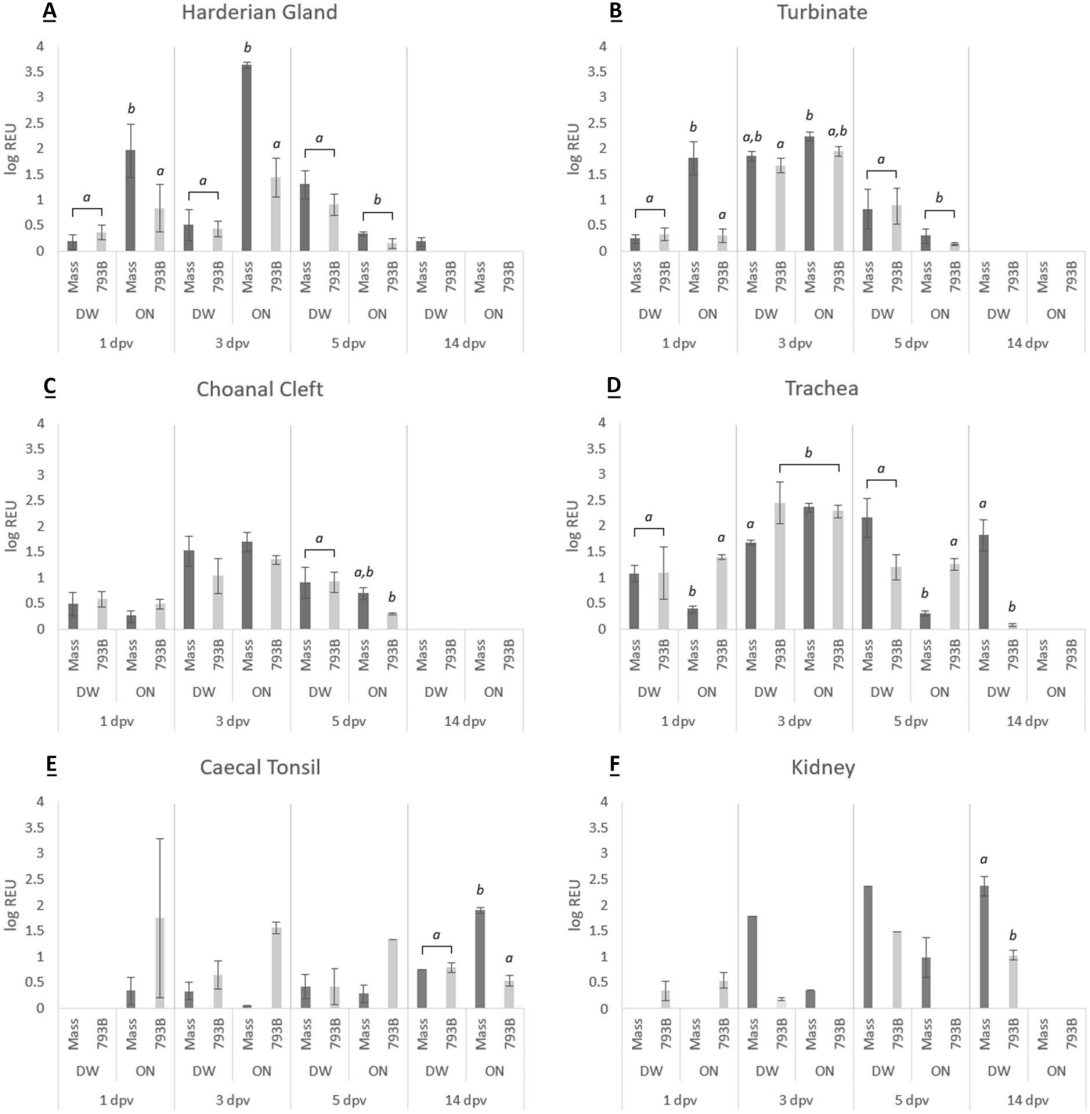
**Quantification of viral RNA expressed as a log REU of RNA in the A HG, B turbinate, C choanal cleft, D trachea, E caecal tonsil and F kidney samples.** Data presented as the mean  ±  SEM. Different letters indicate significant (*p*  < 0.05) differences within time points.

Viral load was significantly higher at 1 and 3 dpv in the ON-Mass group compared to other groups (Figure 6A). Both DW-vaccinated groups had a significantly higher viral load compared to ON-vaccinated groups at 5 dpv (Figure 6A). In the DW-Mass vaccinated group, viral copies were significantly higher at 5 dpv compared to other sampling days. Both 793B vaccinated groups and the ON-Mass group were negative at 14 dpv.

#### Turbinate

Viral RNA load was significantly higher for all groups at 3 dpv in comparison to the other time points, with the exception of ON-Mass. There were significantly higher viral loads in the ON-Mass-vaccinated birds at 1 dpv, compared to all other groups, and in both DW groups compared to the ON-vaccinated group at 5 dpv (Figure 6B).

#### Choanal cleft

Viral load was significantly higher at 3 dpv than all the other sampling days in the Mass-vaccinated groups and the ON-793B group. Significantly higher levels were noted at 5 dpv in both DW-vaccinated groups compared to ON-793B birds (Figure 6C).

#### Trachea

Viral copy numbers were significantly higher at 3 dpv for all groups, compared to other time points, with the exception of DW-Mass, which was significantly higher at 5 dpv. In addition, the ON-Mass birds were significantly lower at 1 and 5 dpv compared to other groups (Figure 6D).

#### Caecal tonsil

The viral load was significantly higher in the ON-Mass group at 14 dpv compared to all other groups (Figure 6E). No further significant differences were noted.

#### Kidney

For 793B, both DW and ON groups were IBV-positive at 1 dpv, whereas only the DW-793B group was positive at 3, 5 and 14 dpv. The Mass groups were only IBV positive from 3 dpv, with the Mass-ON group negative at 14 dpv. In addition, the Mass-DW group was significantly higher than the 793B-DW group at 14 dpv (Figure 6F).

### Differential mRNA expression: TLR3 and MDA5

#### Harderian gland (HG)

**Figure 7:**
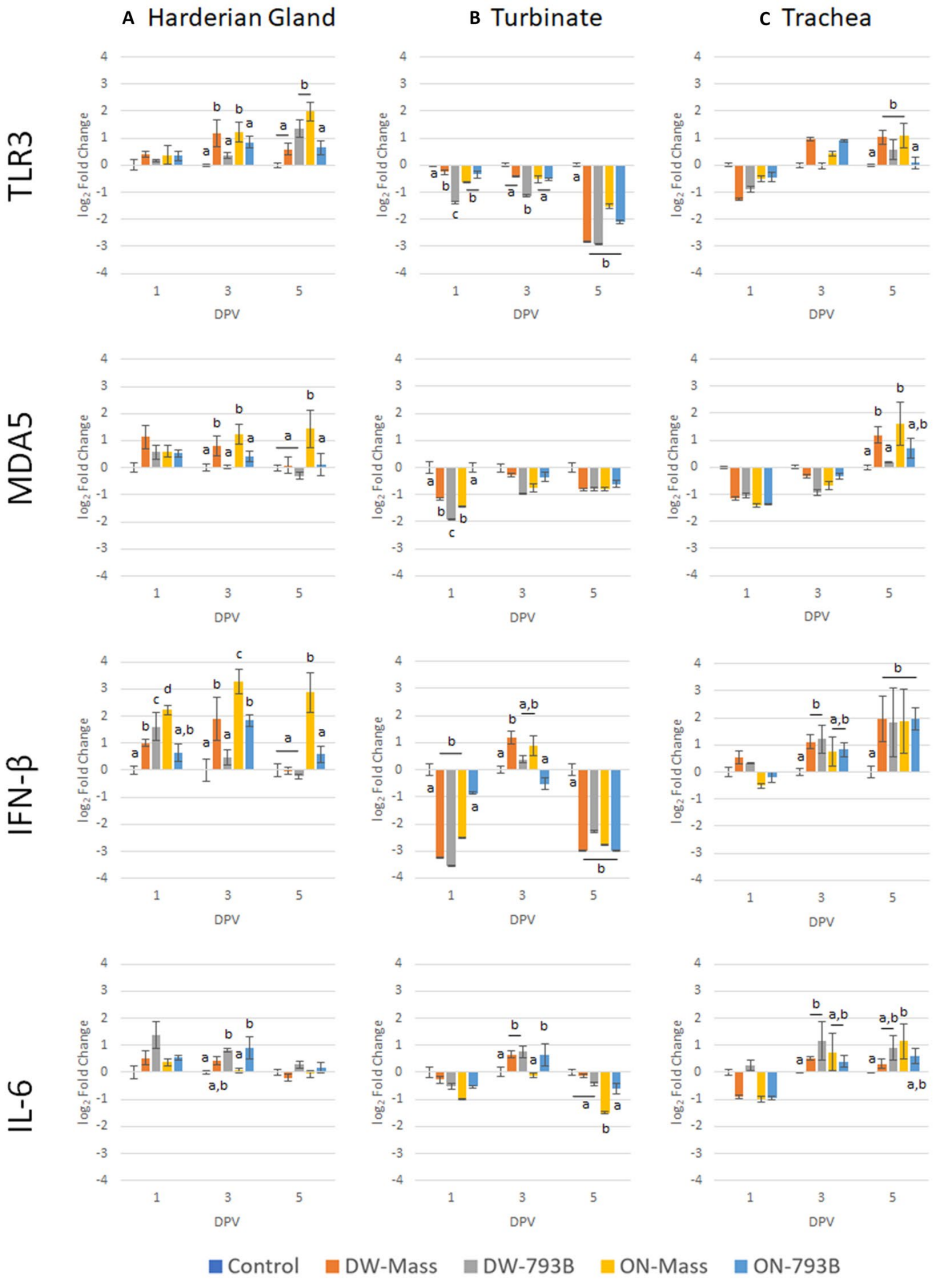
**Expression profile of host genes in Harderian gland, turbinate, choanal cleft and trachea tissue following IBV Mass or IBV 793B vaccination of layer chickens.** Data is shown as the log_2_ fold change when compared to the non-vaccinated group. Different letters indicate significant differences (*p*  < 0.05) in fold change between vaccine strains and vaccination route on the same sample day.

There was significant up-regulation in the mRNA expression of TLR3 in Mass-vaccinated birds via both routes of vaccination at 3 dpv, and at 5 dpv in DW-793B and ON-Mass birds compared to other groups (Figure 7). Expression of MDA5 was significantly up-regulated at 3 and 5 dpv in ON-Mass birds compared to other groups, with the exception of the DW-Mass group at 3 dpv.

#### Turbinate

Significant down-regulation was noted in TLR3 mRNA expression at 1 and 3 dpv in DW-793B vaccinated chickens, compared to all other groups. This was followed by significant down-regulation in all groups at 5 dpv. There was significant down-regulation of MDA5 transcription at 1 dpv in the DW-793B and Mass-vaccinated birds.

#### Trachea

There was significant up-regulation of TLR3 mRNA expression at 5 dpv in both Mass-vaccinated and DW-793B groups. Measurement of MDA5 transcription showed significant up-regulation at 5 dpv in Mass-vaccinated chickens via both immunisation routes (Figure 7).

### Differential mRNA expression: IFN-β

#### Harderian gland (HG)

There was significant up-regulation of IFN-β at all-time points in ON-Mass-vaccinated birds, and at 1 and 3 dpv in DW-Mass-vaccinated birds. Significant up-regulation of IFN-β mRNA expression was observed in the DW-793B vaccinated group at 1 dpv, and at 3 dpv in the ON-vaccinated groups (Figure 7).

#### Turbinate

There was significant down-regulation of IFN-β mRNA expression in both Mass vaccinated groups and in the DW-793B group at 1 dpv, followed by significant down-regulation in all groups at 5 dpv. Significant up-regulation of IFN-β mRNA was observed in the DW-Mass group at 3 dpv.

#### Trachea

Expression of IFN-β mRNA was significantly up-regulated in both DW vaccinated groups at 3 and 5 dpv and in all vaccinated groups at 5 dpv.

### Differential mRNA expression: IL-6

#### Harderian gland (HG)

There was significant up-regulation of IL-6 mRNA expression at 3 dpv in the 793B-vaccinated groups compared to other groups, with the exception of DW-Mass (Figure 7).

#### Turbinate

At 3 dpv, IL-6 was significantly up-regulated in the DW-Mass and both 793B-vaccinated groups. At 5 dpv, there was significant down-regulation of IL-6 mRNA expression in the ON-Mass group.

#### Trachea

Expression of IL-6 mRNA was significantly up-regulated in ON-Mass (5 dpv) and DW-793B (3 dpv) vaccinated chickens (Figure 7).

### Cell-mediated immune responses: mRNA expression of CD8-α and CD8-β

#### Turbinate

**Figure 8:**
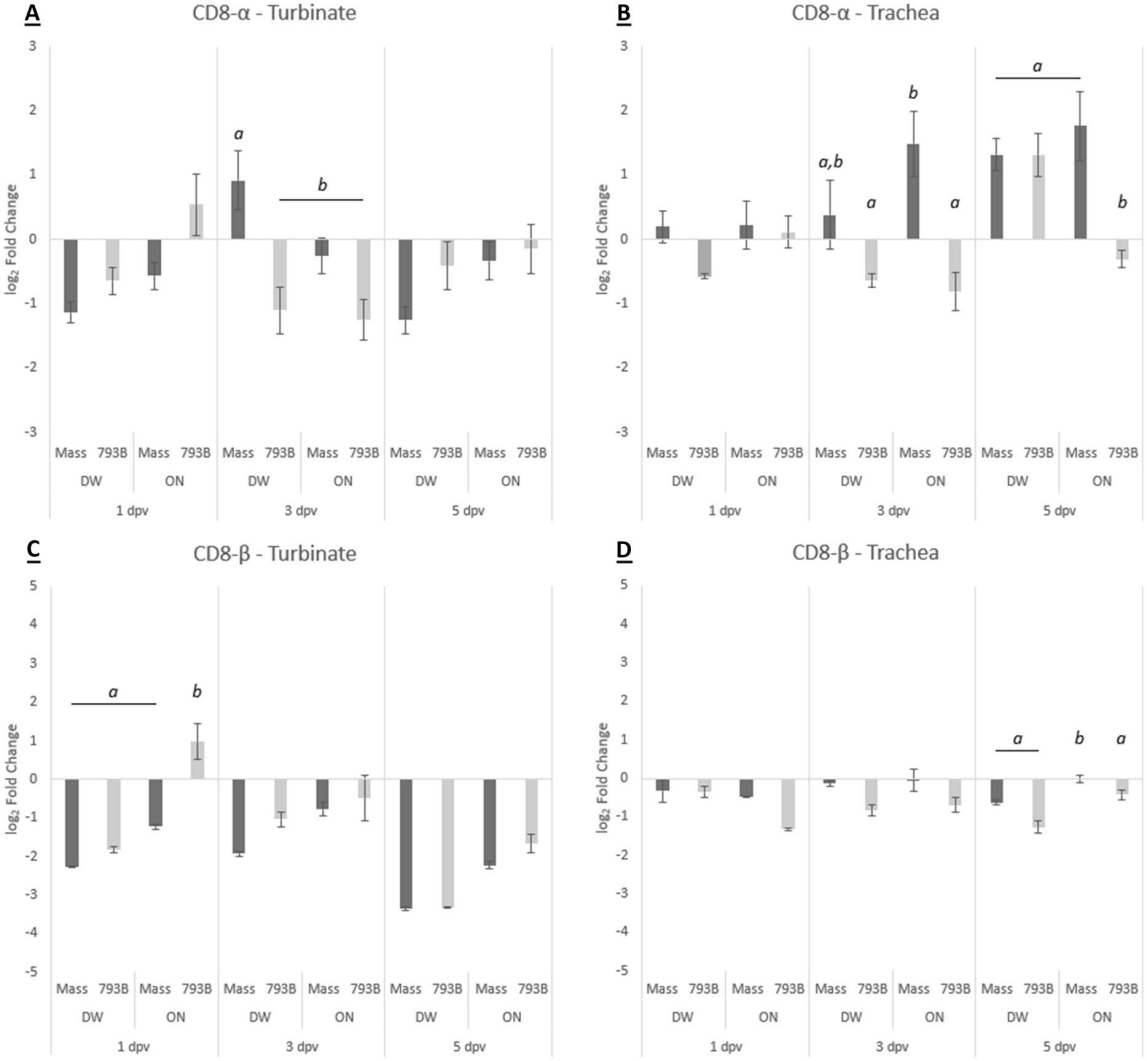
**Relative mRNA expression of A CD8-α in turbinate tissue, B CD8-α in trachea tissue, C CD8-β in turbinate tissue and D CD8-β in trachea tissue at 1, 3, and 5 dpv.** Data are expressed as log_2_ mean fold change compared to the control group  ±  SEM. Expression is calculated based on double delta Ct (ΔΔCt) values. Different letters indicate significant differences (*p*  < 0.05) within time points, determined using one-way ANOVA.

Expression of CD8-α and CD8-β mRNA was significantly down-regulated in all vaccinated groups on the majority of sampling days. In contrast, CD8-α mRNA expression was significantly up-regulated at 3 dpv in the DW-Mass group compared to other vaccinated groups (Figure 8). Both 793B-vaccinated groups were significantly down-regulated at 3 dpv compared with the control, whereas the DW-Mass group was down-regulated at 1 and 5 dpv. For CD8-β, mRNA expression was significantly greater in the ON-793B group compared to all other groups at 1 dpv. All groups were significantly down-regulated compared to the control, with the exception of ON-793B at 1 and 3 dpv.

#### Trachea

Significant up-regulation of CD8-α was noted at 3 dpv in the ON-Mass group compared to both 793B-vaccinated groups (Figure 8). At 5 dpv, the ON-793B group was significantly down-regulated compared to all other vaccinated groups. All groups at 5 dpv, with the exception of ON-793B, were significantly up-regulated compared to the control. Levels of CD8-β transcripts in the DW-Mass, DW-793B and ON-793B group was significantly down-regulated at 5 dpv compared to the ON-Mass group. The ON-793B group was significantly down-regulated compared to the control at 1 and 3 dpv, whereas the DW-793B group was down-regulated at 3 and 5 dpv.

## Discussion

Live attenuated IBV vaccines have been widely used in young commercial broiler [45] and rearing layer/breeder chickens [46], and are normally not administered in hens in lay. Despite this, in recent years, the “off-licence” use of live IBV vaccines in egg-laying birds has increased in many countries. In the literature, we found no information on viral loads of infectious bronchitis vaccine viruses or immune responses in head-associated lymphoid and respiratory tissues of commercial egg-laying hens. To address this, we investigated the viral load, innate, mucosal, and cellular immune responses following vaccination via two different routes—oculonasal and drinking water. We compared responses following vaccination via the oral route to those vaccinated via the oculonasal route, as individual dosing of birds through the respiratory route often gave optimal immune responses and protection against a number of IBV challenge viruses [18]. Globally, an increasing number of producers are administering live IBV vaccines via drinking water [47], as it is convenient for farm workers to prepare and administer the vaccine and as it is less stressful to the birds compared to the intranasal or intraocular routes due to reduced animal handling. Drinking water administration is also presumed to cause less irritation to the respiratory mucosa, and normally has little to no impact on egg production [48].

To diagnose IBV, swab samples (OP and/or CL) and tissues are often examined for the virus, either by RT-PCR, qRT-PCR or virus isolation. In this study, following ON vaccine administration, the viral load of individual OP, CL and tissue samples were examined at intervals by qRT-PCR. From OP swabs, the Mass vaccine was detected for a shorter period (up to 7 dpv) compared to 793B, which persisted up to 14 dpv. In contrast, for the CL swabs, Mass vaccine was detected earlier, but infrequently (1, 5 and 14 dpv), compared to 793B which was only detected at 7–14 dpv. The earlier detection of Mass vaccine, followed by later detection of 793B vaccine viruses, has been reported previously in broiler chickens [49]. Following ON administration, viral loads for both strains were quantified in HG, turbinate, choanal cleft, trachea, caecal tonsil and kidney tissues, however, levels differed depending on tissue type. Significantly higher viral loads of Mass vaccine were often found in the HG, turbinate, caecal tonsil and kidneys. Interestingly, in the trachea, a higher 793B viral load was found at 1 and 5 dpv, with neither strain detected at 14 dpv. It appears that in most tissues, the Mass vaccine was able to establish itself more efficiently than 793B when administered by the ON route. Following DW application, IBV detection and Mass and 793B vaccine viral loads in OP swabs were similar to ON administration. In contrast, for CL swabs, Mass and 793B vaccines viruses were persistently detected at higher levels compared to ON application. This may indicate potential virus replication in the gastrointestinal and/or renal tissues. Interestingly, following DW application, no significant differences in viral loads between each vaccine were seen in the HG, turbinate, choanal cleft or caecal tonsil. For the trachea, the 793B vaccine had a significantly higher viral load at 3 dpv, whereas the Mass vaccine showed significantly higher levels at 5 and 14 dpv. For kidney tissues, both vaccines persisted up to 14 dpv, however, Mass was significantly higher than 793B at 3, 5 and 14 dpv. Overall, it appears that with DW application, the Mass vaccine disseminated slowly to tissues, and persisted at higher viral loads in the trachea and kidneys, when compared to 793B.

All birds in this study received live IB Mass vaccine at day-old, an inactivated IB 793B vaccine at 14-days-old, and a M41 + D274 vaccine at 15–18 weeks of age [15–17]. No other live or killed IBV vaccines were administered after the birds were transferred into an "all-in all-out" free-range layer farm. Following ON vaccination of either strain, significantly higher levels of humoral antibodies were found at 14 dpv in both vaccinated groups (Mass—8306.4 and 793B—7998.8) compared to the control (4062). In contrast to ON vaccination, when the vaccines were given by DW, antibody levels in the 793B group (8075) were significantly higher than the Mass group (5464.6) at 14 dpv. In egg-laying hens, it has been shown that serum anti-IBV antibody levels have a significant influence in providing protection against a drop in egg production and quality [19, 46].

For mucosal immunity, immunoglobulin A (IgA) antibodies play an important role in conferring protection against IBV, and the levels of IBV-specific IgA in lachrymal fluid have been reported before [53–56]. In this study, mucosal immunity was assessed by quantifying IgA and IgY antibodies in lachrymal fluid. No significant increase in IBV-specific IgA was found in any of the ON-vaccinated groups. In contrast, lachrymal anti-IBV IgA levels in the Mass vaccine group given via DW had a significant increase at 14 dpv. Previous studies have reported increased levels of IgA in lachrymal fluid following ocular and/or DW vaccination of day-old SPF or broiler chicks [14, 57, 58]. High levels of IBV-specific IgA have also been associated with a degree of protection against virulent IBV [14]. For IgY (the equivalent of IgG in mammals), detection in lachrymal fluid could have been due to transudation [59] and/or local secretion by B-cells [60]. In the current study, it appears that oculonasal inoculation of either vaccine resulted in a significant increase in lachrymal IgY by 14 dpv. This is potentially due to greater IBV replication and inflammation in the upper respiratory and lymphoid tissues, including the HG [61]. These results are similar to those found by Gallego et al. who demonstrated that soluble antigen administration in seven-week-old chickens via the ocular route is an efficient application for producing a local immune response in the HG [62]. The significantly higher levels of lachrymal IgY at 14 dpv also corresponded with the overall increase of humoral IBV antibody titres [13, 14, 51], reflecting increased transduation of IgY into the lacrymal fluid. This appears to be particularly true for the 793B vaccine, as we only detected significant IgY levels in the DW-793B group, and not for the DW-Mass group. The local memory immune response has been shown to be dominated by IgY [13]. Therefore, higher levels of IgY in egg-laying hens would be beneficial in minimising IBV infection, subsequently avoiding or reducing egg production losses, and sustaining a good overall flock health.

Although indirect ELISAs (as described above) are preferred for measuring mucosal immunity, it is not always possible for several reasons. This includes the requirement of IgA/IgY monoclonal antibodies, IBV antigen purification and standardization, the small quantity of lachrymal fluid which is collectable from each bird, the laborious process of tracheal wash collection, and the inclusion of upper respiratory tissues (e.g., turbinate) where no fluid could be collected. For these reasons, we also attempted to measure the regulation of IgA and IgY mRNA transcription in tissues of turbinate and trachea. This allowed for quantification of mucosal immunity parameters in tissue samples. For changes in IgA or IgY mRNA transcription, comparisons between the ON-vaccinated groups showed up or down regulation depending on the vaccine strain and sampling timepoint. For turbinate tissues of Mass-vaccinated hens, IgA and IgY mRNA was down-regulated at all sampling points, whereas those vaccinated with 793B showed a significant up-regulation at 3 (IgA and IgY) and 5 (IgA) dpv. In contrast, for the trachea, up-regulation of IgA mRNA, but not IgY, was found for both Mass or 793B vaccinated hens. Interestingly, up-regulation of IgA mRNA in the Mass group was significantly higher than the 793B group at 5 dpv. This may suggest consistent and higher replication of both Mass and 793B in the trachea, whereas the turbinate appears to support better replication of 793B. This was consistent with viral load data in turbinate and trachea tissue as described above. In contrast to ON application, IgA mRNA expression in the trachea and turbinate following DW administration showed down-regulation at all sampling points. This could have been due to slow dissemination of the vaccines and/or lower viral load in the HG and respiratory tissues. The early up-regulation of IgY mRNA at 3 dpv in the turbinate and trachea appears to be associated with the peak of 793B viral load. Also, an increase of IL-6 was noted at the same sampling point, indicating increased inflammation.

Early immune responses following virulent IBV infection or vaccination in young SPF and broiler chicks have been published before [36, 63, 64]. In this study, for the first time, certain parameters of the early immune response in Mass or 793B vaccinated egg-laying hens are reported. Findings showed that mRNA transcription of TLR3 was significantly up-regulated in the HG (3–5 dpv) and trachea (5 dpv) following either ON or DW inoculation. Similarly, MDA5 was significantly increased in the HG and trachea. Up-regulated transcription of both genes has been previously demonstrated in the respiratory tissues of SPF and broiler chicks [61, 65]. Significantly higher transcripts in Mass-vaccinated hens in contrast to the 793B groups, reflects a potentially slower 793B dissemination and replication, and recognition by the host [66]. In contrast, for DW application, differences in magnitude or pattern were not consistently noted in the HG, turbinate or trachea between Mass and 793B groups. This could have potentially been associated with a lower viral load in these tissues when either vaccine was given by DW in comparison to ON.

Although cell-mediated immunity (CMI) is an essential part of the immune response to IBV, there is no information available on the CMI responses following live IB vaccination in egg-laying hens. In this study, following ON application, the Mass vaccine produced a stronger induction of CMI compared to the 793B vaccine, as demonstrated by significantly raised CD8-α transcripts in the trachea at 3–5 dpv. This is likely due to the Mass vaccine virus’s ability to disseminate and replicate more efficiently in respiratory tissues. It has been reported that CD8 + cells expressing the CD8-α chain are involved in elimination of IBV-infected cells during early infection [57, 67, 68]. Significant up-regulation (compared to the control) of CD8-α mRNA in the trachea of the Mass ON group at 3–5 dpv, compared to only 5 dpv for 793B, again reflects the ability of the Mass vaccine to replicate and stimulate local cellular immune responses. Based on this study, it appears that by 5 dpv, the host cellular response to Mass vaccination, either given by DW or ON, were comparable.

In conclusion, this study represents the first investigation to evaluate the viral load of Mass or 793B attenuated live vaccines, and host immune responses to these vaccines, in egg-laying hens. This study has demonstrated that the pattern of IBV replication differs according to tissue type, vaccine strain, and inoculation route. For both vaccines, the dissemination and clearance of the vaccine viruses were slower when given by DW compared to the ON route. When given by the ON route, both vaccines were able to induce comparable levels of mucosal immunity. The Mass IBV vaccine induces cellular immunity to similar levels regardless of vaccination method. When given either by ON or DW, the 793B vaccine induced significantly higher levels of humoral immunity. These findings can be used for improved IB vaccination strategies in egg-laying hens to prevent drop in egg production and/or quality.
